# Imatinib attenuates cardiac fibrosis by inhibiting platelet-derived growth factor receptors activation in isoproterenol induced model

**DOI:** 10.1371/journal.pone.0178619

**Published:** 2017-06-01

**Authors:** Le-Xun Wang, Xiao Yang, Yuan Yue, Tian Fan, Jian Hou, Guang-Xian Chen, Meng-Ya Liang, Zhong-Kai Wu

**Affiliations:** 1 Second Department of Cardiac Surgery, The First Affiliated Hospital, Sun Yat-sen University, Guangzhou, China; 2 Assisted Circulatory Laboratory of Health Ministry, The First Affiliated Hospital, Sun Yat-sen University, Guangzhou, China; 3 Key Laboratory of South China Agricultural Plant Molecular Analysis and Genetic Improvement, South China Botanical Garden, Chinese Academy of Sciences, Guangzhou, China; 4 Guangdong Provincial Key Laboratory of Applied Botany, South China Botanical Garden, Chinese Academy of Sciences, Guangzhou, China; University of Otago, NEW ZEALAND

## Abstract

Cardiac fibrosis is a significant global health problem with limited treatment choices. Although previous studies have shown that imatinib (IMA) inhibited cardiac fibrosis, the anti-fibrotic mechanisms have not been clearly uncovered. The aim of this study is to evaluate whether IMA attenuates cardiac fibrosis by inhibiting platelet-derived growth factor receptors (PDGFR) on isoproterenol (ISO)-induced mice. Adult male C57BL/6 mice were treated with vehicle or ISO ± IMA for one week. After echocardiography examination, the hearts of mice were used for histopathologic, RT-qPCR, and western blot analyses. We found that the ventricular wall thickness, cardiac hypertrophy, and apoptosis were enhanced following ISO treatment. IMA decreased the left ventricular wall thickness, prevented hypertrophy, and inhibited apoptosis induced by ISO. In addition, IMA attenuated the accumulation of collagens and α-smooth muscle actin (α-SMA) (the markers of fibrosis) caused by ISO treatment. Moreover, the expression of fibrosis related genes, and the phosphorylation of PDGFRs in ISO-treated mice hearts were inhibited by IMA as well. However, IMA did not change the expression of the matrix metalloproteinase-9 (MMP-9) in ISO-treated hearts. Furthermore, IMA reduced the expressions of collagens as well as α-SMA caused by activation of PDGFRα in cardiac fibroblasts. Taken together, our data demonstrate that IMA attenuated the cardiac fibrosis by blocking the phosphorylation of PDGFRs in the ISO-induced mice model. This study indicates that IMA could be a potentially therapeutic option for cardiac fibrosis in clinical application.

## Introduction

Cardiac fibrosis, one of the common pathological manifestations following many cardiovascular disease conditions such as cardiac surgery, myocardial ischemia, myocardial infarction and chronic hypertrophy induced by pressure overload, is characterized by the excessive production and deposition of the extracellular matrix (ECM) proteins in heart, and seriously affects the prognosis of patients [1–3]. Various reports have shown that cardiac fibroblasts play the important roles in the occurrence, the development and the outcome of cardiac fibrosis [2, 4–8]. Aldosterone, angiotensin II (Ang II), transforming growth factor-β1 (TGF-β1), platelet-derived growth factors (PDGFs), endothelin-1 (ET-1) and tumor necrosis factor-α (TNF-α) are known to promote the fibroblast activation and cardiac fibrosis [1, 9–11]. After binding the ligands (PDGFs and TGF-β1), tyrosine kinase receptors (PDGFRs and TGF-β1 receptor) are activated and play the core role in the cardiac fibrosis [1]. However, the definite mechanisms responsible for cardiac fibrosis have not been uncovered. Therefore, there are currently no effective therapies that can prevent its occurrence or halt its progression.

Imatinib mesylate (Imatinib, IMA), a small molecule inhibitor of tyrosine kinase (TKI), has been approved for the treatment of BCR-ABL positive leukemia and gastrointestinal stromal tumors (GIST), which has dramatically improved the clinical outcome of those cancers [12, 13]. IMA inhibits not only BCR-ABL/c-Abl and c-Kit kinases activation but also other tyrosine kinase, such as PDGFRs [14]. The potential treatment effect of IMA in non-malignant diseases such as fibrosis has been paid more attention. The anti-fibrotic effect of IMA has been demonstrated in pulmonary fibrosis, liver fibrosis, scleroderma fibroblasts, and renal fibroblasts [14–17]. IMA could attenuate cardiac fibrosis in spontaneously hypertensive rat model [18], desoxycorticosterone induced salt-sensitive hypertensive rat model [19], and myocardial infarction model [20]. The mechanism of IMA in different model is diverse.

Chronic stimulation by catecholamines such as isoproterenol (ISO) in animal models is known to induce cardiac hypertrophy, fibroblast activation, and fibrosis [10, 21–23]. However, the anti-fibrotic effect of IMA in ISO-induced cardiac fibrosis has not been investigated. The aim of this study was to evaluate the effect of IMA in ISO-induced cardiac fibrosis mice model. Then we examined whether IMA inhibited PDGFRs tyrosine kinase activity in attenuating cardiac fibrosis *in vivo* and *in vitro*.

## Materials and methods

### Reagents

Isoproterenol (ISO) was purchased from Sigma-Aldrich (St. Louis, MO, USA) and dissolved in PBS. Imatinib (IMA) was obtained from MedChem Express (Monmouth Junction, NJ, USA) and dissolved in PBS. Phenylmethanesulfonyl fluoride (PMSF) was purchased from Sigma-Aldrich. Recombinant murine Platelet-Derived Growth Factor-AA (PDGF-AA) was obtained from PeproTech (Rocky Hill, NJ, USA).

### Animal and treatments

Adult male C57BL/6 mice (11–13 weeks old) were divided into 4 groups: vehicle, ISO, IMA, IMA plus ISO. The vehicle group was treated with PBS. Cardiac fibrosis was induced by subcutaneous ISO injection daily (20 mg/kg/d for 7 days) [24–26], and/or IMA (40 mg/kg/d for 7 days) [27, 28] was administered by intraperitoneal injection daily. After 7 days, animal echocardiography was used to preliminarily evaluate the success of the cardiac fibrosis model. At the end of the study mice were anesthetized with 3% isoflurane and a terminal blood sample was drawn immediately from the left ventricle. Blood was centrifuged and serum was stored at -80°C. Euthanasia was performed by cervical dislocation under deep anesthesia with isoflurane at day 8, and hearts were excised and frozen in liquid N_2_ and stored at -80°C until biochemical analysis or fixed in paraformaldehyde for histological analysis. No mice died before the end of the experiment. All animals used in the present study were purchased from the animal center of Sun Yat-sen University and raised carefully in accordance with the Guide for the Care and Use of Laboratory Animals (2011). All experimental procedures were approved by the Animal Care and Use Committee of Sun Yat-sen University (Permit Numbers: SCXK (Guangdong) 2015–0107).

### Echocardiography

Mice were anaesthetized with 1.5% isoflurane/oxygen, and cardiac function was assessed using transthoracic echocardiography (VisualSonics system, Toronto, Ontario, Canada) performed at day 8. M-mode and two-dimensional echocardiography were performed to assess cardiac parameters, including left ventricular (LV) end-diastolic dimension, wall thickness, LV fractional shortening and ejection fraction, whilst pulse-wave Doppler was used to assess mitral valve flow (E/A ratio), as reliable measures of diastolic function.

### Immunoassay for serum cardiac troponin T (cTnT)

Serum cTnT was assayed by enzyme-linked immunosorbent assay (ELISA) using mouse immunoassay kits from Uscn Life Science Inc. (Wuhan, China), according to the protocol.

### Real-time quantitative polymerase chain reaction (RT-qPCR)

Total RNA was extracted from the ventricle of heart or cells by TRIzol reagent (Invitrogen), and cDNA was synthesized using the qPCR RT Master Mix kit (TOYOBO, Osaka, Japan). PCR primers were designed and synthesized by Invitrogen (Shanghai, China), as illustrated in Table 1. Quantitative PCR analysis was performed according to the instructions using a KOD SYBR qPCR Mix (TOYOBO) by LightCycle 480 (Roche, Basel, Switzerland). For analysis, the expression of target genes was normalized to the GAPDH.

**Table 1:** Primer sequences used for RT-PCR.

Gene (Mouse)	Forward	Reverse
GAPDH	GGTCATCCATGACAACTT	GGGGCCATCCACAGTCTT
collagen I	AACTCCCTCCACCCCAATCT	CCATGGAGATGCCAGATGGTT
collagen III	ACGTAAGCACTGGTGGACAG	GGAGGGCCATAGCTGAACTG
PDGF-A	AGCGTCAAGTGCCAGCCTTC	CTCACCTCACATCTGTCTCCTCCT
PDGF-B	GGGTGAGCAAGGTTGTAATG	AAGGAAGTGGAGGCAATGGACAG
PDGF-C	GCTGCTGATGCTGGCTATGGT	GATTGACTCCTCTTGGTGCCTCTG
PDGF-D	TGACATGGTGGCTCCGTTCC	TCCTCTGACAACAGTGCTGCTCTC
PDGFRα	CAACCACACTCAGACGGATG	GCGGCAAGGTATGATGGCAGAG
PDGFRβ	GCGACACTCCAACAAGCAT	TGTAGCCACCGTCACTCTC
TGF-β1	ACCGCAACAACGCCATCTAT	TTCAGCCACTGCCGTACAACTC
CCN2	TGTCTTCGGTGGGTCGGTGT	CAGGCAGTTGGCTCGCATCATAG
HGF	TCAGCACCATCAAGGCAAGG	GCACATCCACGACCAGGAACAAT
MMP-9	AACCACAGCCGACAGCACCT	ATCCAGTACCAACCGTCCTTGAAG

### Western blot analysis

Proteins were isolated from the ventricular homogenate or cells with lysis buffer (Beyotime Institute of Biotechnology, Shanghai, China) with PMSF. Equal amounts of protein were subjected to SDS-PAGE and transferred to PVDF membranes (Milliopore, Billerica, MA, USA). The membranes were blocked and then incubated with GAPDH (Proteintech, Rosemont, IL, USA), MMP-9, α-SMA, p-PDGFRα (Tyr720), PDGFRα (Abcam, Cambridge, MA, USA), p-PDGFRβ (Tyr740), PDGFRβ (Cell Signaling Technology, Danvers, MA, USA), PDGF-A, PDGF-B, PDGF-C and PDGF-D (Bioss, Beijing, China). Subsequently, the membranes were incubated with an HRP-conjugated secondary antibody (Thermo Fisher Scientific, Waltham, MA, USA) at room temperature for 1 h and were visualized using enhanced chemiluminescence reagents (Sigma-Aldrich) according to the manufacturer’s instruction.

### Cell culture

Cardiac fibroblasts were harvested from adult C57BL/6 mice and cultured as reported previously [29]. Briefly, hearts were removed and washed with PBS. After enzymatic digestion by 0.1% Collagenase II (Gibco, South Logan, UT, USA), cardiac fibroblasts were cultured in Dulbecco's modified Eagle's medium (DMEM, Gibco) supplemented with 100 U/ml penicillin, 100 ug/ml streptomycin and 10% fetal bovine serum (FBS, Gibco). After three passages, cells were collected and passaged for further experiments. All cells cultures were maintained at 37°C in an atmosphere of 5% CO_2_. After three passages, cardiac fibroblasts were cultured in presence PDGF-AA (10 ng/ml) for 24 h, and the cells were harvested for protein expression assays by western blotting or mRNA assays using RT-qPCR.

### Histological analysis

Excised hearts were fixed in 4% paraformaldehyde, paraffin embedded, and sectioned at 5 μm thickness. Deparaffinized sections were stained for Hematoxylin-Eosin (H&E) or picrosirius red staining. Image-Pro Plus software (Media Cybernetics, Rockville, MD, USA) was used to measure fibrosis from 10 random fields per section. FITC was used to measure apoptosis in deparaffinized heart sections via terminal deoxynucleotidyl-transferase-mediated dUTP nick-end labeling (TUNEL, Roche). Deparaffinized sections were incubated with proteinase K, and DNA strand breaks were labeled according to manufacturer’s instructions. Cells were visualized at the Zeiss microscope (Carl Zeiss, Jena, Germany) and the percentage of TUNEL-positive nuclei calculated from 10 random fields per section.

### Immunohistochemistry (IHC)

Ki-67 or α-SMA was detected in the ventricle of mice heart by IHC. After ethanol exposure and hydration, the sections were rinsed in PBS, quenched for 10 min in methanol containing 3% H_2_O_2_, and incubated for 15 min in blocking solution (PBS containing 2% BSA and 0.1% Triton X-100), followed by incubation overnight in primary antibodies against Ki-67 (Sigma-Aldrich, diluted 1:50) or α-SMA (Abcam, diluted 1:400) in blocking solution. After washing with PBS, the sections were incubated for 60 min with the secondary antibody (Goat anti-rabbit IgG, (H+L), horseradish peroxidase conjugated, Thermo Fisher Scientific). DAB substrate kit for peroxidase was then used to stain sections as described in the manufacturer`s instructions (Sigma-Aldrich). The sections were counterstained with hematoxylin. The images were acquired using a Zeiss microscope. The expression region of Ki-67 or α-SMA was quantified and analyzed using the Image-Pro Plus software.

### Statistical analysis

Data were presented as mean ± SEM. Statistical analysis was performed using GraphPad Prism Software (Version X, La Jolla, CA, USA) and SPSS v. 16.0 (SPSS, Inc. Armonk, NY, USA). The ANOVA with Tukey’s multiple comparisons test (equal variance) or the Kruskal-Wallis test, followed by Dunn’s Multiple Comparison test (unequal variance) was used for multiple comparisons. *p*< 0.05 was considered statistically.

## Results

### IMA decreased the left ventricular (LV) wall thickness in ISO-induced mice model

**Fig 1:**
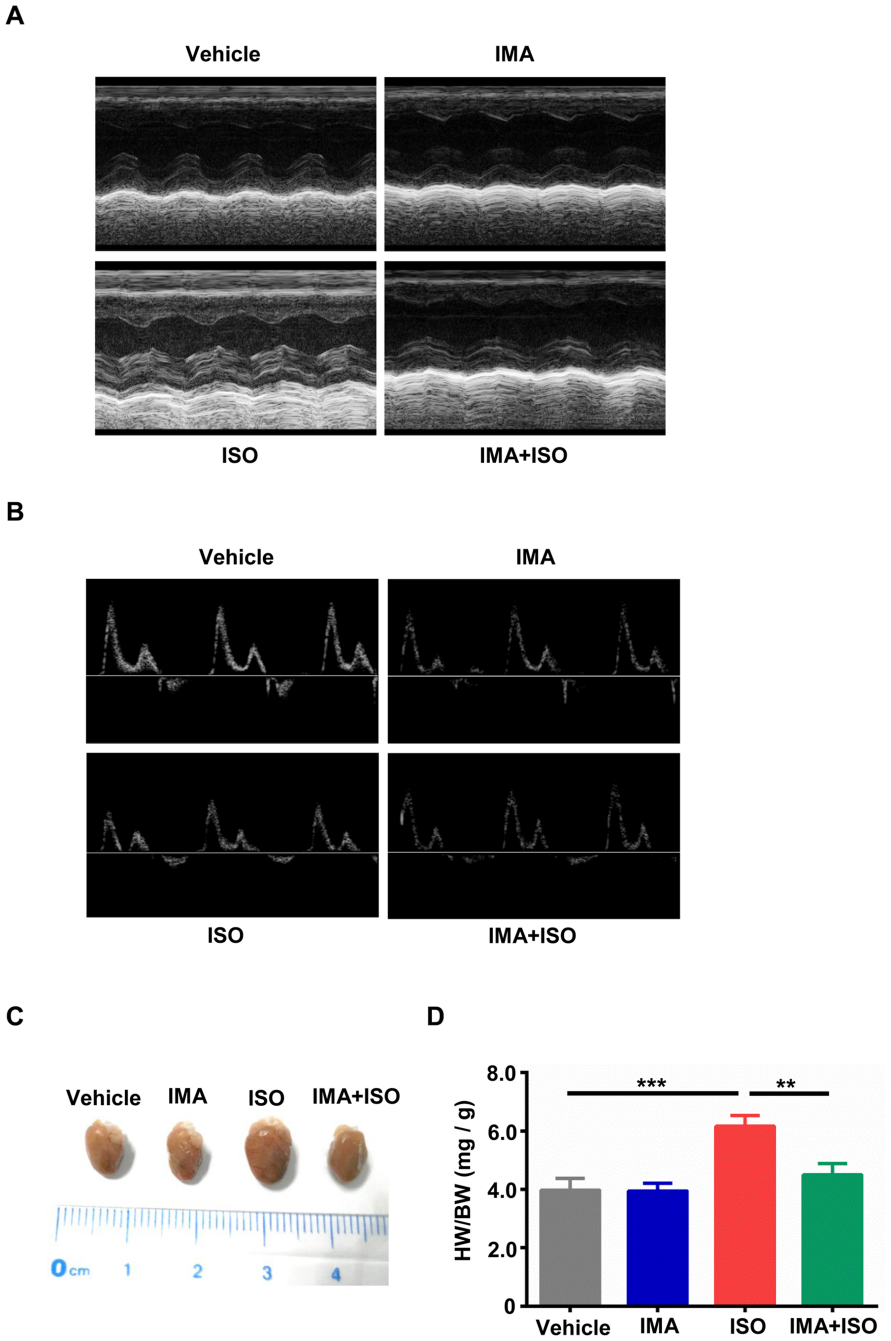
IMA reduces the ventricular wall thickness and cardiac hypertrophy caused by ISO. Mice were treated with vehicle, Imatinib (IMA, 40mg/kg/d, injected intraperitoneally everyday), Isoproterenol (ISO, 20mg/kg/d, injected subcutaneously everyday), IMA + ISO for 7 days. (A) The ventricular wall thickness was detected by M-mode echocardiography at day 8. (B) The early to late diastolic peak velocity (E/A) ratio was detected by Doppler echocardiography at day 8. (C) Hearts removed from one mouse in each group are shown. (D) The gravimetric analysis of heart weight to body weight (HW/BW) ratio. (n = 5–8 per group, **: *p*<0.01, ***: *p*<0.001).

To assess changes of the cardiac structure and function in response to catecholamine or/and IMA stimulation, adult male C57BL/6 mice were treated with vehicle, ISO (20mg/kg/d, injected subcutaneously everyday), IMA (40mg/kg/d, injected intraperitoneally everyday), IMA + ISO for one week. Cardiac function of the mice was assessed using echocardiography. As shown in Fig 1A, treatment with ISO increased the LV wall thickness in comparison with that of vehicle treatment. Compared to ISO-treated group, IMA + ISO-treated group was showed to decrease the LV wall thickness significantly (Fig 1A and Table 2). The LVIDd was significantly lower in the ISO group than that in the IMA + ISO, IMA and vehicle groups (Table 2). Combined IMA + ISO treatment prevented ISO-mediated increase in heart rate (HR), IVSd, IVPWd, IVSs, and IVPWs, whereas IMA did not induce changes in cardiac function on its own compared to vehicle (Table 2). No significant differences were found in LV percent fractional shortening (FS), percent ejection fraction (EF), E/A ratio, LVEVd, LVEVs, and LVIDs among the vehicle, IMA, ISO and IMA + ISO groups (Fig 1B and Table 2). These results suggested that IMA improved left ventricular structure in ISO-induced mice model.

**Table 2:** Echocardiographic parameters.

Variables	Vehicle	IMA(40mg/kg/d)	ISO(20mg/kg/d)	IMA+ISO
HR	456±31	463±24	558±31 *&	485±25
IVSd(mm)	0.60±0.04	0.68±0.06	0.87±0.05 *&	0.74±0.04 *
LVIDd(mm)	4.21±0.15	4.10±0.15	3.42±0.11 *&	3.86±0.06 *
LVPWd(mm)	0.63±0.06	0.62±0.04	0.85±0.07 *&	0.75±0.04
LVEVd(μL)	37.57±2.12	36.58±3.02	34.43±2.51	35.28±2.07
IVSs(mm)	0.91±0.06	0.94±0.06	1.27±0.09 *&	1.05±0.10
LVIDs(mm)	3.17±0.11	3.05±0.10	2.79±0.12	2.88±0.13
LVPWs(mm)	1.01±0.11	1.21±0.18	1.75±0.06 *&	1.36±0.17
LVEVs(μL)	16.77±1.15	16.21±2.41	14.52±3.45	14.97±2.12
FS,%	32.37±1.92	33.57±2.25	28.28±2.15	30.47±2.28
EF, %	60.36±3.89	56.77±5.03	53.21±4.17	53.72±4.85
E, mm/s	564±33	543±40	464±28	546±41
A, mm/s	271±21	261±43	229±26	267±35
E/A ratio	2.08±0.04	2.10±0.19	2.03±0.13	2.05±0.11

Data were expressed as mean ± SEM. HR: Heart Rate; IVSd: end-diastolic interventricular septal thickness; IVSs: end-systolic interventricular septal thickness; LVIDd: end-diastolic left ventricular internal diameter; LVIDs: end-systolic left ventricular internal diameter; LVPWd: end-diastolic left ventricular posterior wall thickness; LVPWs: end-systolic left ventricular posterior wall thickness; LVEVd: end-diastolic left ventricular volume; LVEVs: end-systolic left ventricular volume; FS: fractional shortening; EF: Ejection Fraction; E: peak early diastolic flow; A: peak late diastolic flow; E/A ratio: left ventricular early to late diastolic peak velocity ratio. (n = 10–13 per group).

*: *p*<0.05 compared with vehicle;

^&^: *p*<0.05 compared with IMA+ISO. IMA: imatinib; ISO: isoproterenol

### IMA inhibits ISO-induced cardiac hypertrophy but has no obvious harm to mice

Previously report showed that ISO could induce the cardiac hypertrophy [30]. We assessed the changes of hearts weight among four groups. The hearts of ISO-treated group were bigger than that in vehicle-treated group and IMA + ISO-treated group (Fig 1C). Heart weight to body weight ratio was significantly lower in the IMA + ISO group than that in the ISO group (*p*<0.01) (Fig 1D). No significant difference in the heart weight to body weight ratio was found among the vehicle, IMA, and IMA + ISO groups (Fig 1D).

We also assessed the safety of treatment with ISO ± IMA on mice. The serum cTnT did not change in any of the groups (Fig A in S1 Fig). The body weights of the mice were stable, with no significant differences among the vehicle-, IMA -, ISO- and IMA + ISO-treated groups (Fig B in S1 Fig). Motor activity and feeding behavior were all normal.

### Effects of IMA on the heart cells survival

**Fig 2:**
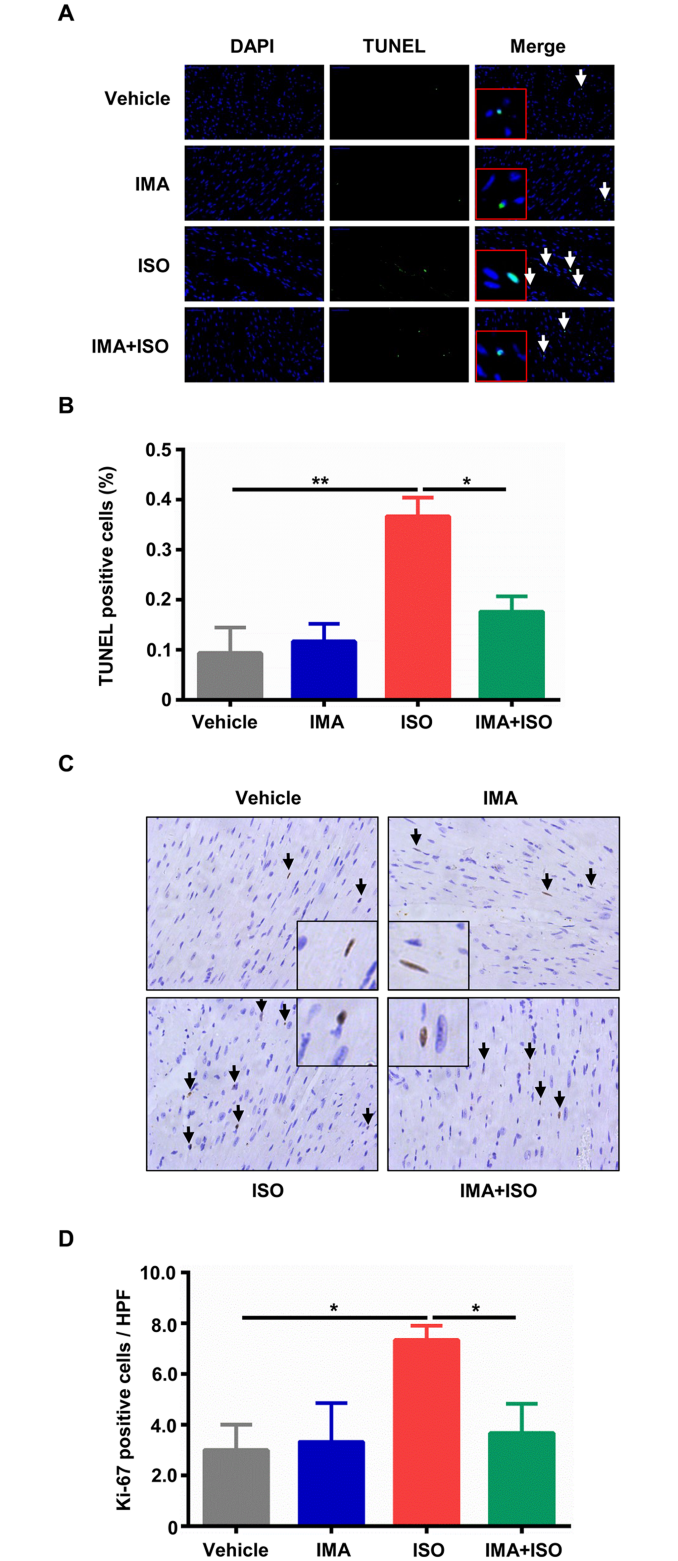
Effects of IMA on the heart cells survival. (A) (B) Representative transferase-mediated dUTP nick-end labeling (TUNEL) staining (400×), and percentage of TUNEL positive cells in the hearts sections from mice treated with vehicle, IMA, ISO, IMA + ISO for one week are shown. Ki-67 was tested by immunohistologic analysis (400×). (C) (D) The percentage of Ki-67 positive cells in the hearts sections from mice treated with vehicle, IMA, ISO, IMA + ISO for one week are shown. (n = 5–8 per group, *: *p*<0.05, **: *p*<0.01). HPF: High Power Field.

Next, we examined the cells apoptosis and proliferation in heart of model mice. Apoptosis, as estimated via TUNEL-positive nuclei was significantly increased at one week following ISO treatment compared to vehicle treatment (*p*<0.01), and IMA treatment prevented ISO-induced apoptosis (*p*<0.05) (Fig 2A and 2B). In ISO-treated group, the number of Ki-67 proliferating interstitial cells was increased compared with vehicle group (Fig 2C and 2D). IMA inhibited the increase of proliferation induced by ISO (*p*<0.05) (Fig 2C and 2D).

### IMA inhibits ISO-induced cardiac fibrosis

**Fig 3:**
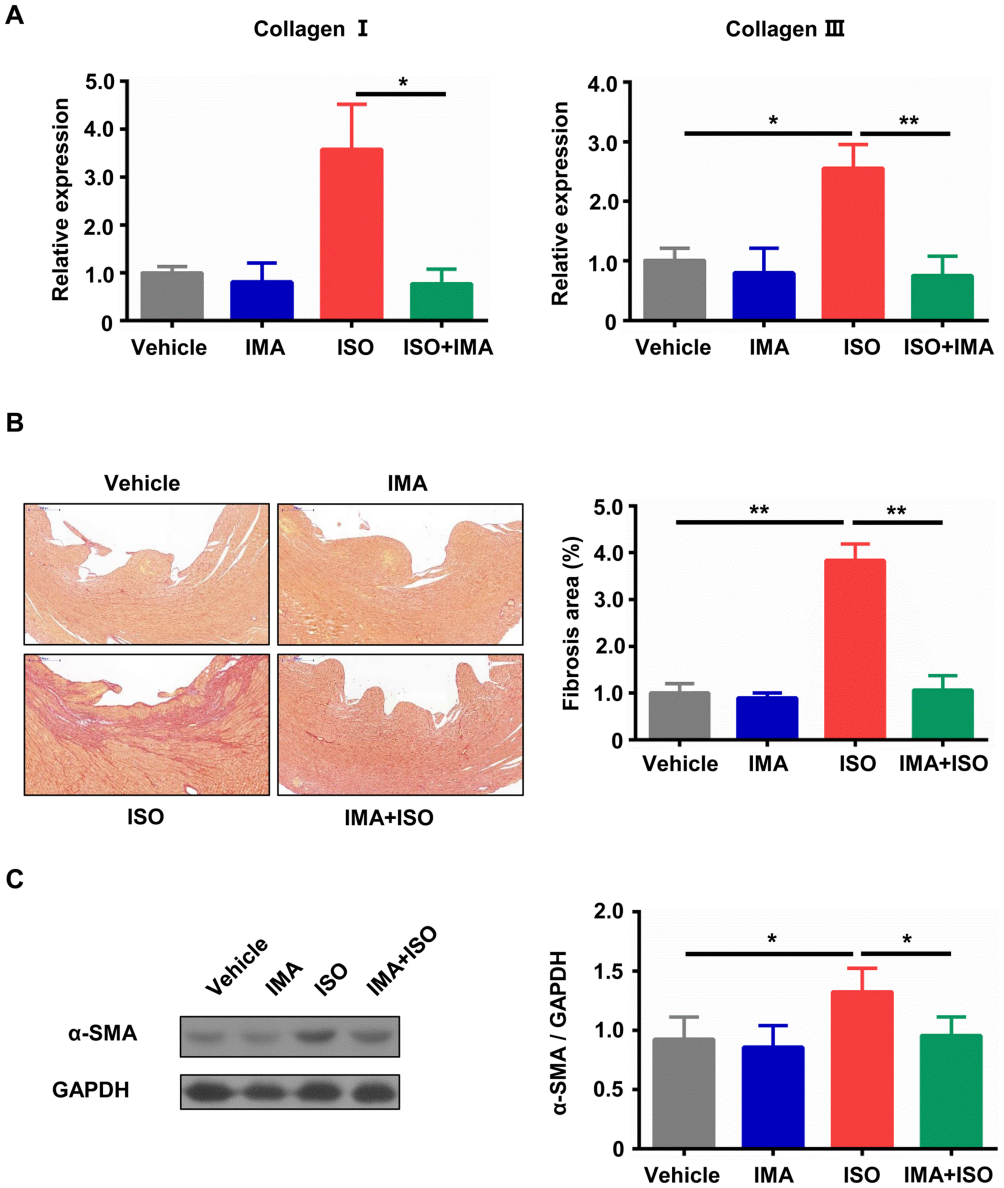
IMA inhibits ISO-induced cardiac fibrosis. (A) Myocardial mRNA expression of collagen I and III was decreased in mice hearts treated with IMA + ISO compared with that in ISO treated mice hearts. (B) Histopathological features of collagen deposition by Sirius red staining of heart sections from mice model (100×), and quantification of sirius red staining. (C) The lysates of hearts tissue from mice treated with vehicle, IMA, ISO, IMA + ISO for one week were subjected to western blotting to analyze the expression of α-SMA, and quantitative analysis of the α-SMA expression. (n = 8 per group, *: *p*<0.05, **: *p*<0.01). (The Kruskal-Wallis test, followed by Dunn’s Multiple Comparison test, was used to perform statistical comparison for mRNA expression of collagen I.).

In addition to hypertrophy, cardiac fibrosis was estimated by RT-qPCR and sirius red staining. As shown in Fig 3A, ISO treatment significantly increased the mRNA levels of collagen I and III when compared with vehicle treatment, and IMA attenuated the increases of collagens caused by ISO. Heart sections were stained with sirius red and observed under a light microscope. Sirius red staining showed striking collagen deposition in the ventricular wall from ISO-treated mice compared with vehicle-treated animals (Fig 3B). Quantitative histology analysis demonstrated that interstitial fibrosis was increased in ISO-treated group when compared with that in vehicle-treated group (*p*<0.01), and it was decreased in IMA + ISO-treated group when compared with that in ISO-treated group (*p*<0.01) (Fig 3B).

Then we tested the protein expression of α-SMA in hearts using western blotting assay. As shown in Fig 3C, ISO treatment significantly increased the level of α-SMA compared with vehicle treatment (*p*<0.05), and the level of α-SMA was decreased in IMA + ISO-treated group (*p*<0.05). The immunohistochemistry result of α-SMA in hearts sections was similar to result of western blot (S2 Fig).

### IMA reduces the expression of fibrosis related genes induced by ISO

**Fig 4:**
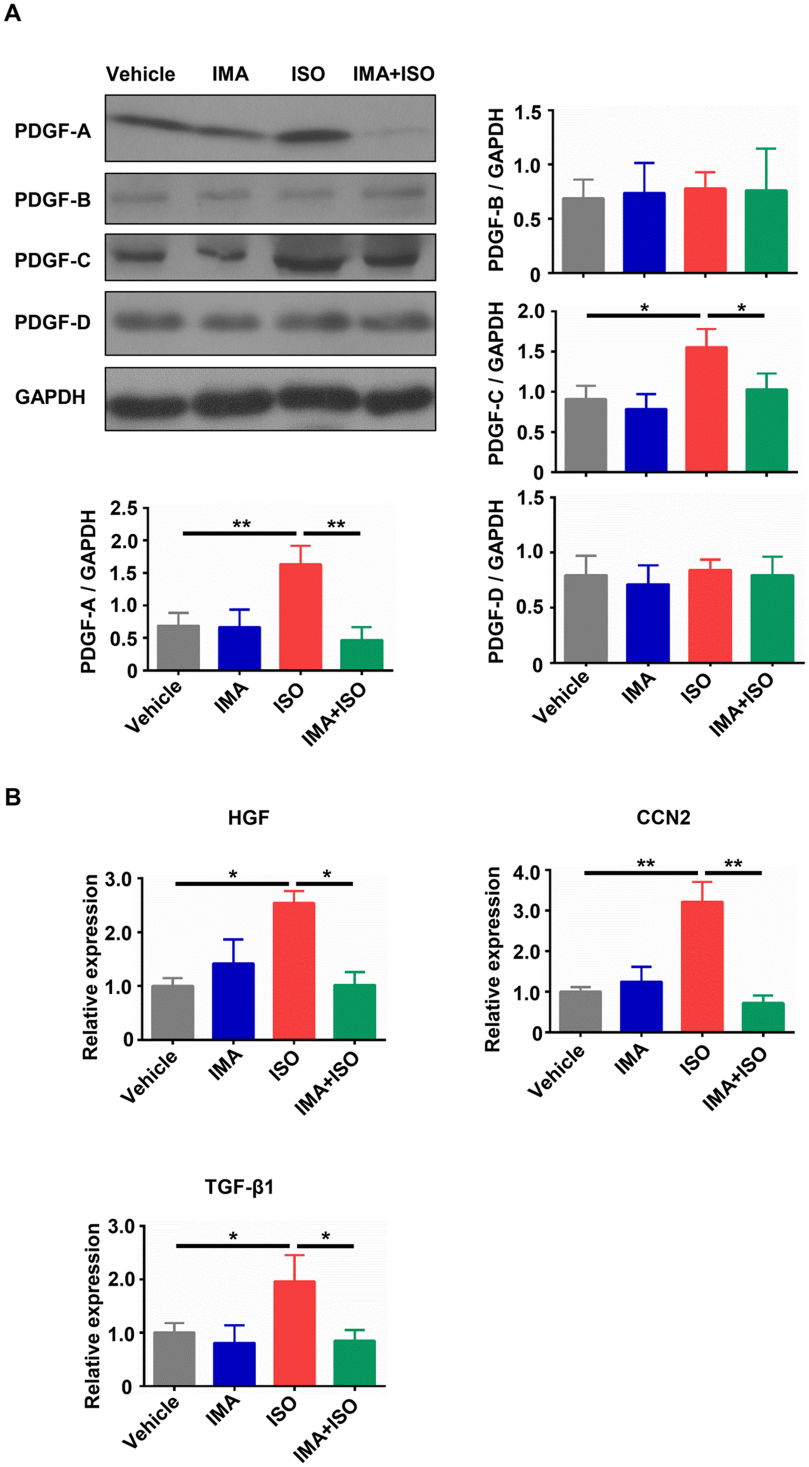
IMA reduces the expression of fibrosis related genes induced by ISO. (A) The protein expression of PDGF-A, PDGF-B, PDGF-C, and PDGF-D in hearts from mice treated with vehicle, IMA, ISO, IMA + ISO for one week was tested by Western blot. (B) The mRNA level of HGF, CCN2, and TGF-β1 in hearts from mice treated with vehicle, IMA, ISO, IMA + ISO for one week was tested by RT-qPCR. (n = 5–8 per group, *: *p*<0.05, **: *p*<0.01).

Due to the crucial role of PDGFs in fibrosis, we examined the protein changes of PDGFs in model mice hearts. The myocardial protein expressions of PDGF-A, PDGF-B, PDGF-C and PDGF-D were examined by Western blot. As shown in Fig 4A, treatment with ISO significantly increased the protein levels of PDGF-A and PDGF-C when compared with vehicle treatment ((*p*<0.01 for PDGF-A; *p*<0.05 for PDGF-C), and the protein levels of PDGF-A and PDGF-C were decreased in IMA + ISO-treated mice hearts compared to that in ISO-treated hearts (*p*<0.01 for PDGF-A; *p*<0.05 for PDGF-C). The levels of PDGF-B and PDGF-D did not significantly change in any of the treatment groups (Fig 4A). The mRNA result of PDGFs in hearts was similar to result of western blot (S3 Fig). Then we tested the mRNA changes of two important molecules associated with fibrosis: connective tissue growth factor (CTGF, also named CCN2) and hepatocyte growth factor (HGF). They were significantly increased in ISO group when compared with that in vehicle group and decreased by IMA + ISO treatment (Fig 4B).

TGF-β1 is known to play a major role in the fibrotic processes [31]. The mRNA change of TGF-β1 in mice hearts was examined. ISO treatment significantly upregulated the TGF-β1 mRNA expression when compared with vehicle treatment and IMA + ISO treatment (*p*<0.05), and there was no significant difference between vehicle-treated group and IMA + ISO-treated group (Fig 4B).

### IMA inhibits the kinase activation of PDGFRs *in vivo*

**Fig 5:**
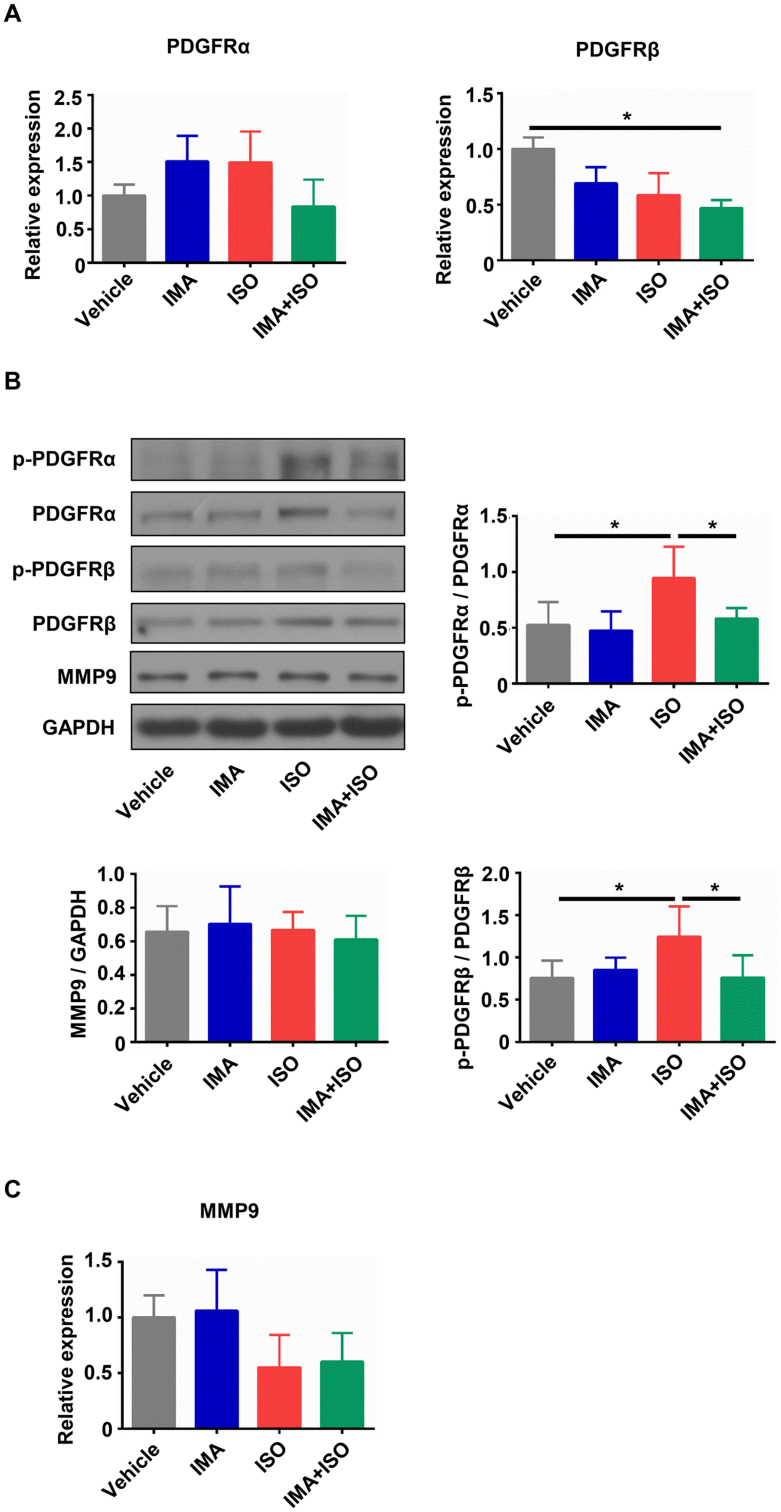
IMA inhibits the kinase activation of PDGFRs in mice heart tissue. (A) The mRNA expressions of PDGFRα and PDGFRβ in hearts from mice treated with vehicle, IMA, ISO, IMA + ISO for one week were tested by RT-qPCR. (B) The lysates of hearts tissue were subjected to western blotting to analyze the phosphorylation level of p-PDGFRα (Tyr720), and p-PDGFRβ (Tyr740), and the expression of PDGFRα, PDGFRβ, and MMP-9. The western results from one mouse in each group and statistical analysis of western blot bands are shown. (C) The mRNA expression of MMP-9 in hearts from four groups was tested by RT-qPCR. (n = 5–8 per group, *: *p*<0.05).

PDGFs exert their biological effects through binding and activation of two receptor tyrosine kinases, PDGFRα and PDGFRβ [32]. Due to the important role of PDGFs/PDGFRs signaling pathway in the fibrosis [33], the changes of PDGFRα and β in mice hearts were tested. RT-qPCR results showed that there were no significant differences in mRNA expression of PGDFRα among four groups (Fig 5A). Compared with other treatments, ISO treatment did not significantly change the mRNA expression of PGDFRβ (Fig 5A). To assess the myocardial phosphorylation state of PDGFRα and PGDFRβ (activated form), we performed western blotting for testing the phosphorylated form (p-PDGFRα: Tyr720; p-PDGFRβ: Tyr740). As shown in Fig 5B, the myocardial phosphorylation of the PDGFRα and PDGFRβ was increased in ISO-treated group compared with that in vehicle group (*p*<0.05), but it was significantly inhibited by IMA + ISO treatment compared to ISO treatment (*p*<0.05).

Due to MMP-9 participation in the degradation of ECM components [34], we assessed the change of MMP-9 in the hearts of model mice. There were no obvious changes of MMP-9 in the mRNA expression and the protein level among the vehicle-, IMA-, ISO- and IMA + ISO-treated groups (Fig 5B and 5C).

### IMA inhibited the expressions of fibrosis related genes by blocking the phosphorylation of PDGFRα in PDGF-AA treated cardiac fibroblasts

**Fig 6:**
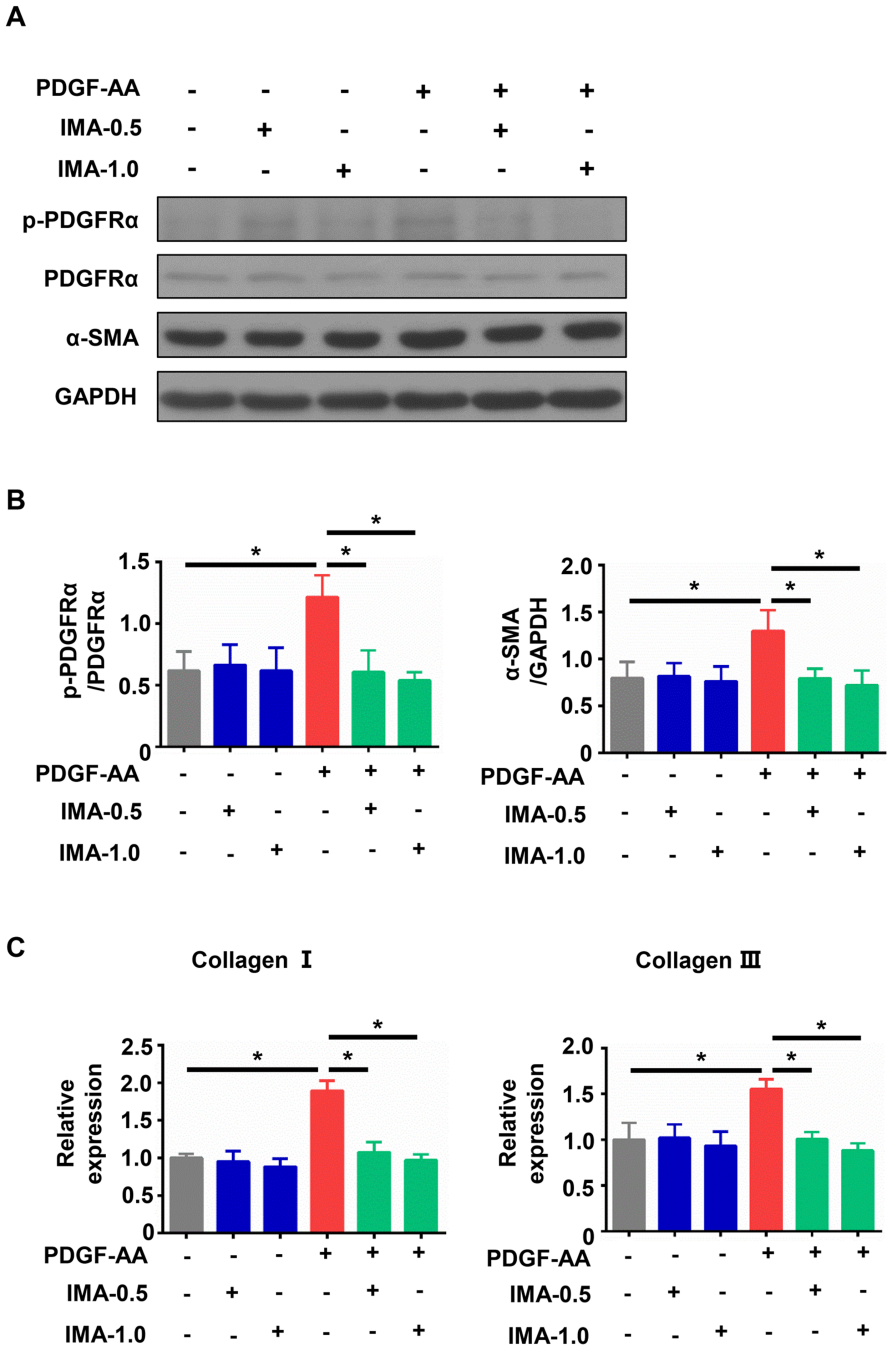
Effect of IMA in PDGF-AA treated mice cardiac fibroblasts. Mice cardiac fibroblasts were treated with PDGF-AA (10 ng/ml), IMA-0.5 (0.5 μM), IMA-1.0 (1.0 μM), PDGF-AA + IMA-0.5, and PDGF-AA + IMA-1.0 for 24 h. (A) The lysates were subjected for western blotting analysis the expression of p-PDGFRα, PDGFRα, and α-SMA. (B) Quantitative analysis of p-PDGFRα and α-SMA protein level. (C) The mRNA expression of collagen I and III. The data are representative of three independent experiments. (*: *p*<0.05).

PDGF-A and phosphorylated PDGFRα were increased in the ISO-treated group (Figs 4A and 5B). As the PDGF-AA isoform only effectively activates PDGFRα [35], we treated mice cardiac fibroblasts with IMA (0.5 μM and 1.0 μM) or/and PDGF-AA (10ng/ml) for 24 h. As shown in Fig 6A and 6B, PDGF-AA treatment increased the phosphorylation of PDGFRα in cultured cardiac fibroblasts. IMA administration blocked the phosphorylation of PDGFRα caused by PDGF-AA. The expressions of α-SMA, collagen I and III were upregulated by PDGF-AA treatment, and were decreased by IMA + PDGF-AA treatment (Fig 6B and 6C).

## Discussion

Cardiac fibrosis is one of significant global health problems and associates with nearly all forms of heart disease [1]. Myocardial infarction, cardiac surgery, pressure overload, cardiomyopathy, toxic factors (such as anthracyclines or alcohol), metabolic disturbances (such as obesity and diabetes), and aging are associated with the development of cardiac fibrosis [36–39]. However, the molecular mechanism underlying cardiac fibrosis has not been fully understood. Therefore, there are currently no efficient therapies available to reverse or arrest the fibrosis.

In this study, we evaluated the anti-fibrotic effect of IMA in ISO-induced cardiac fibrosis mice model. Our findings showed that treatment with 40 mg/kg of IMA decreased the LV thickness, and attenuated the cardiac hypertrophy in the ISO-induced mice model. Treatment with IMA inhibited the apoptosis and proliferation in the ISO-treated heart cells. IMA attenuated the accumulation of interstitial collagens and α-SMA, and down-regulated the increases of fibrosis related genes in the ISO-induced mice model. The blunting of collagens synthesis caused by IMA was associated with decreased phosphorylation of PDGFRα and β. Moreover, inhibition of PDGFRα by IMA decreased the mRNA expressions of collagen I and III and the protein level of α-SMA in PDGF-AA-treated mice cardiac fibroblasts. Thus, IMA attenuated the cardiac fibrosis by inhibiting the kinase activation of PDGFRs.

Cardiac fibroblast is widely accepted to be responsible for cardiac fibrosis. TGF-β1 and PDGFs are key driving forces in fibroblasts activation [1]. TGF-β1 binds its receptors and promotes fibrosis by upregulating ECM and tissue inhibitors of matrix metalloproteinase gene expression, as well as suppressing MMP gene level [1]. PDGF-A and PDGF-B induced cardiac fibrosis in transgenic mice by activation of PDGFRα and PDGFRβ, respectively [40]. PDGF-C induced fibroblasts activation, cardiac fibrosis, hypertrophy, and dilated cardiomyopathy through up-regulation and activation of PDGFRα [41]. PDGF-D promoted activation of cardiac fibroblasts by binding PDGFRβ [9, 42]. In myocardial infarction, PDGF-A and PDGF-D were significantly increased in myofibroblasts [43]. PDGFs not only induced the activation of cardiac fibroblast, but also elevated TGF-β1 expression *in vivo* and *in vitro* [9, 20, 42]. In addition, PDGFs also directly stimulate fibroblasts to contract collagens and differentiate into myofibroblasts [17]. In our study, ISO treatment increased the mRNA expressions of PDGF-A and PDGF-C, enhanced the activation of PDGFRα and PDGFRβ, and elevated TGF-β1 expression. However, the PDGF-B and PDGF-D transcriptions were not changed among four groups. These data are in stark contrast to a recent study that PDGF-D was increased in the infarcted heart [43]. Disulfide bridging between PDGF chains results in the formation of the homo-dimeric molecules PDGF-AA, PDGF-BB, PDGF-CC and PDGF-DD or the heterodimeric PDGF-AB molecule [33]. PDGF-AA induces fibroblasts activation through binding and activating PDGFRα [35]. So cardiac fibroblasts stimulated by PDGF-AA were used to explore the mechanism in our study. We found that IMA inhibited the expressions of fibrosis related genes by blocking the phosphorylation of PDGFRα in PDGF-AA treated cardiac fibroblasts.

MMP-9 can cleave ECM proteins and plays an important role in atherosclerosis, hypertension, myocardial infarction, heart failure, and cardiac fibrosis [34]. The MMP-9 protein level was increased in ISO-induced cardiac hypertrophy rat model [44]. In contrast, ISO treatment did not change the mRNA transcription and the protein level of MMP-9 in mice hearts of our model. There may be some reasons: 1) the responses of rat and mouse to ISO may be different at some extent; 2) the dose of ISO was different (rats subcutaneously injected with 170 mg/kg/d ISO, and mice treated with 20 mg/kg/d ISO); 3) the time of testing MMP-9 was different (4 weeks after administration of ISO for 4 days in rat model, after treatment with ISO for 7 days in mice model).

Animal models are used to better understand the pathogenesis and the mechanism of cardiovascular diseases to improve diagnosis, prevention and therapy of cardiac disease and to help develop and test new diagnostic, preventive and therapeutic procedures [45]. Many animal models have been used for the research of cardiac fibrosis, such as spontaneously hypertensive induced cardiac fibrosis model [18], the surgery models (myocardial infarction model, myocardial ischemia/reperfusion injury model, and transverse aortic constriction model) [45], and the induced models (desoxycorticosterone induced salt-sensitive hypertensive model [19], Ang II induced cardiac fibrosis model [46], and ISO induced cardiac fibrosis model [10]). However, the mechanisms of cardiac fibrosis in different models are diverse. Chronic ISO infusion in mice is a common model system that mimics the elevated catecholamines and sustained β-adrenergic receptors (β-AR) stimulation observed during cardiac hypertrophy that lead to fibrosis [47–50]. In our current study, we found that ISO treatment in C57BL/6 mice increased cardiac hypertrophy, cardiomyocyte death, interstitial fibrosis, and cardiac dysfunction. Moreover, the tyrosine kinase receptors of PDGFRs were activated in ISO-treated hearts. Recent studies showed that enhanced expression of β3-adrenoceptor, gefitinib, difluoromethylornithine (DFMO) inhibited the cardiac fibrosis in ISO-induced mice model [10, 30, 50]. In this study, we found that IMA attenuated the cardiac fibrosis induced by ISO. Our results indicated that IMA could be a potential therapeutic approach to prevent cardiac fibrosis in clinical application.

The ways of administration and dose of ISO contribute differently on the survival of myocardial cells. As in previous studies (ISO < 50 mg/kg/d), myocardial apoptosis was observed in ISO-induced animal models [30, 50]. However, myocyte apoptosis was not increased after treatment with single injections of 200 or 300 mg/kg ISO in mice [51]. In our study, adult male C57BL/6 mice were treated with ISO (20 mg/ kg/d) for one week and we found that apoptosis was significantly increased in ISO-treated mice heart cells. In addition, the proliferation and activation of cardiac fibroblast is recognized for its fundamental contributions to the heart’s response to various forms of injury [2]. Our results showed that ISO treatment increased the Ki-67 positive cells in mice hearts. Moreover, the expression of α-SMA, an important marker of myofibroblasts which are activated fibroblasts, was increased in ISO-treated mice hearts. Our data indicated that ISO treatment increased the proliferation of cardiac fibroblasts, and induced the activation and the differentiation of these cells to myofibroblasts. Furthermore, IMA reduced the apoptosis and proliferation induced by ISO in mice heart cells. These results indicated that IMA inhibited the injury of heart and the activation of cardiac fibroblasts caused by ISO.

Many studies have demonstrated the anti-fibrotic effect of IMA in pulmonary fibrosis, renal fibrosis, liver fibrosis and dermal fibrosis [15, 17, 52–54], whereas few studies have investigated the effect of IMA on the heart. Jang et al reported that IMA attenuated cardiac fibrosis and improved diastolic cardiac dysfunction in a hypertensive rat model [18]. In the desoxycorticosterone induced salt-sensitive hypertensive rat model and the myocardial infarction rat model, IMA attenuated cardiac remodeling and reduced myocardial fibrosis [19, 20]. Consistent with these results, our data showed that IMA attenuated myocardial fibrosis and improved cardiac function in the ISO-treated mice. As a classical tyrosine kinase inhibitor, IMA inhibits the kinase activation of PDGFRs, c-Kit and c-Abl [14]. Recent studies demonstrated that the mechanisms of inhibition fibrosis by IMA were associated with blocking the activation of PDGFRs, and c-Abl [18–20]. Our results showed that IMA inhibited the activation of PDGFRα and PDGFRβ and decreased the mRNA expression of TGF-β1.

In a hypertensive rat model, IMA attenuated cardiac fibrosis by inhibiting the activation of PDGFRβ as well as the phosphorylation of c-Abl [18]. In the hypoxia-induced pulmonary arterial hypertension model, IMA inhibited the perivascular fibrosis via blocking PDGFRβ pathway [15]. Liu et al reported that IMA inhibited the activation of PDGFR and reduced the mRNA expression of TGF-β1 in myocardial infarction model [20]. In our study, IMA inhibited not only the activation of PDGFRβ but also the phosphorylation of PDGFRα as well as the up-regulated mRNA of TGFβ1 caused by ISO treatment.

The cardiovascular toxicity issue related to IMA is somewhat controversial. Although all TKIs approved for CML therapy share activity against BCR-ABL, they are distinct in their potency and activity against other kinases, and each TKI has a distinct toxic effect profile [55]. Due to the strong potency binding kinase domain of new-generation tyrosine kinase inhibitors, dasatinib, nilotinib, and ponatinib treatment increased the risk of vascular occlusive events compared with IMA treatment in patients with CML [56]. Although some studies have been reported that longer-term IMA treatment was associated with cardiac toxicity, including asymptomatic LV dysfunction and congestive heart failure in patients with CML or GISTs [57, 58], the true incidence of this side effect is still uncertain. Prospective and sequential cardiac imaging in patients on IMA showed a low incidence of asymptomatic cardiac dysfunction, comparable to the expected population incidence [59, 60]. This indicated that IMA-related pathological changes in the myocardium did not necessarily translate into clinical significant cardiac toxicity, especially under short-term treatment with IMA [61]. In addition, IMA reversed experimentally induced PAH in animal models [62]. Our results showed that IMA treatment did not increase the level of serum cTnT and decrease the weight of mice. Moreover, IMA treatment reduced the apoptosis of heart cells caused by ISO. These results indicated that IMA did not cause cardiotoxicity at normal administered dose and had a protective effect on the heart. In others and our studies, the subjects of observation or experiments did not have cardiac failure before the observation or experiments started. And there are no reports to study the cardiovascular toxicity of IMA in cardiac failure patients or animal models. So the short-term and long-term effect of IMA in patients or animal models with cardiac failure need to be further investigated.

In summary, we showed that IMA attenuated cardiac fibrosis and improved diastolic dysfunction in the ISO-induced mice model by inhibiting the phosphorylation of PDGFRs. Our results provided an approach for treatment cardiac fibrosis with IMA in clinical application.

## Supporting information

S1 Fig Imatinib has not obvious harm to mice. After treatment with vehicle, IMA, ISO, IMA plus ISO for one week, mice were euthanized and the hearts were excised at day 8. (A) The serum cTnT of mice was measured by ELISA. (B) The body weights were monitored and plotted versus time. (n = 8 per group).
(TIF)

S2 Fig Imatinib inhibits the increase of α-SMA induced by ISO in mice hearts. (A) (B) Histopathological feature of α-SMA in hearts was tested by immunohistologic analysis (100×) and quantitative analysis. (n = 8 per group, *: *p*<0.05, **: *p*<0.01).
(TIF)

S3 Fig IMA reduces the mRNA of PDGFs gene induced by ISO. (A)-(D) The mRNA expression of PDGF-A, PDGF-B, PDGF-C, and PDGF-Din hearts from mice treated with vehicle, IMA, ISO, IMA + ISO for one week was tested by Western blot (n = 5–8 per group, *: *p*<0.05, **: *p*<0.01).
(TIF)
