# Supplementary figures and images for: Imatinib attenuates cardiac fibrosis by inhibiting platelet-derived growth factor receptors activation in isoproterenol induced model
Source: PLoS One. 2017 Jun 1;12(6):e0178619. doi: 10.1371/journal.pone.0178619 (PMC5453565; doi:10.1371/journal.pone.0178619)

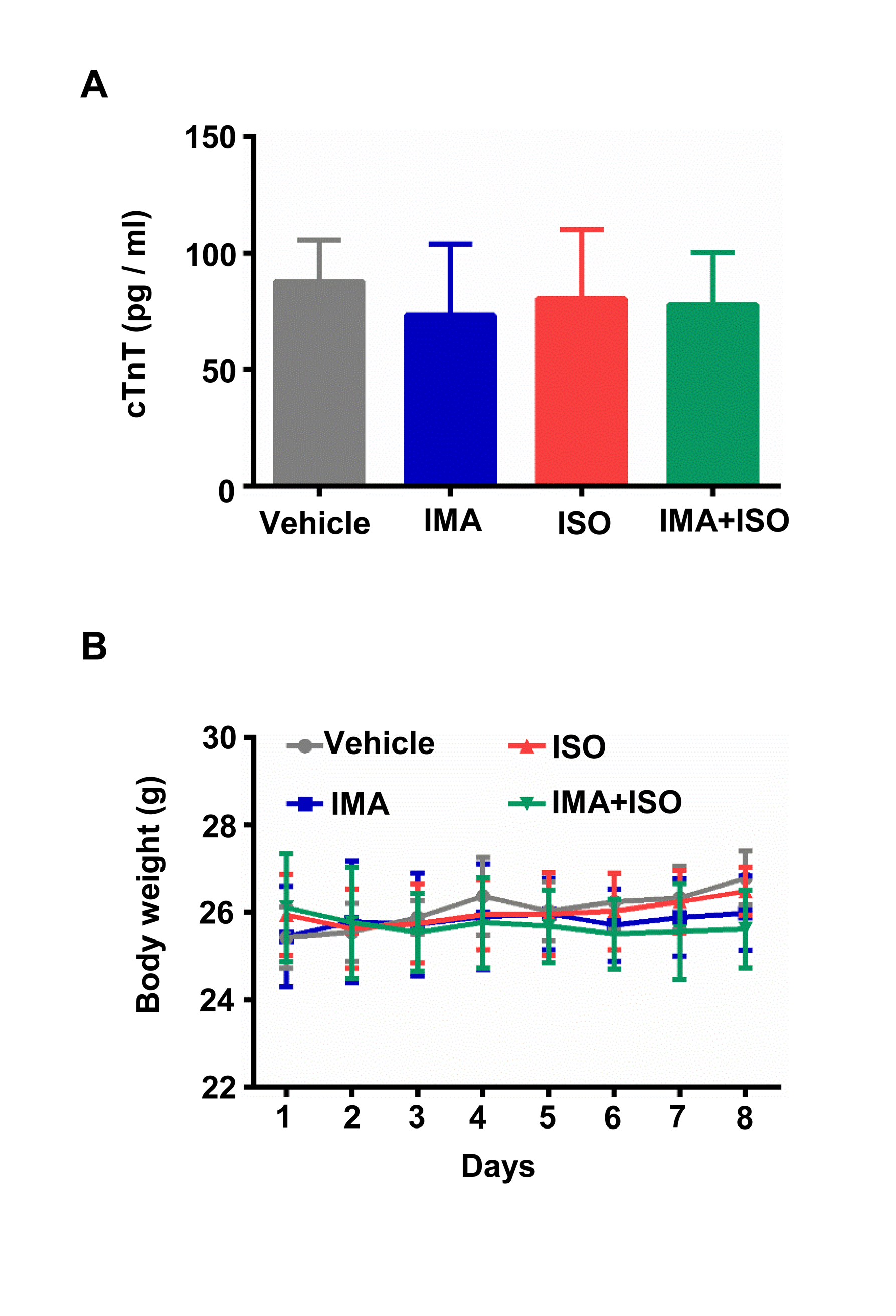

Supplement: S1 Fig — After treatment with vehicle, IMA, ISO, IMA plus ISO for one week, mice were euthanized and the hearts were excised at day 8. (A) The serum cTnT of mice was measured by ELISA. (B) The body weights were monitored and plotted versus time. (n = 8 per group). (TIF) [file pone.0178619.s001.tif]

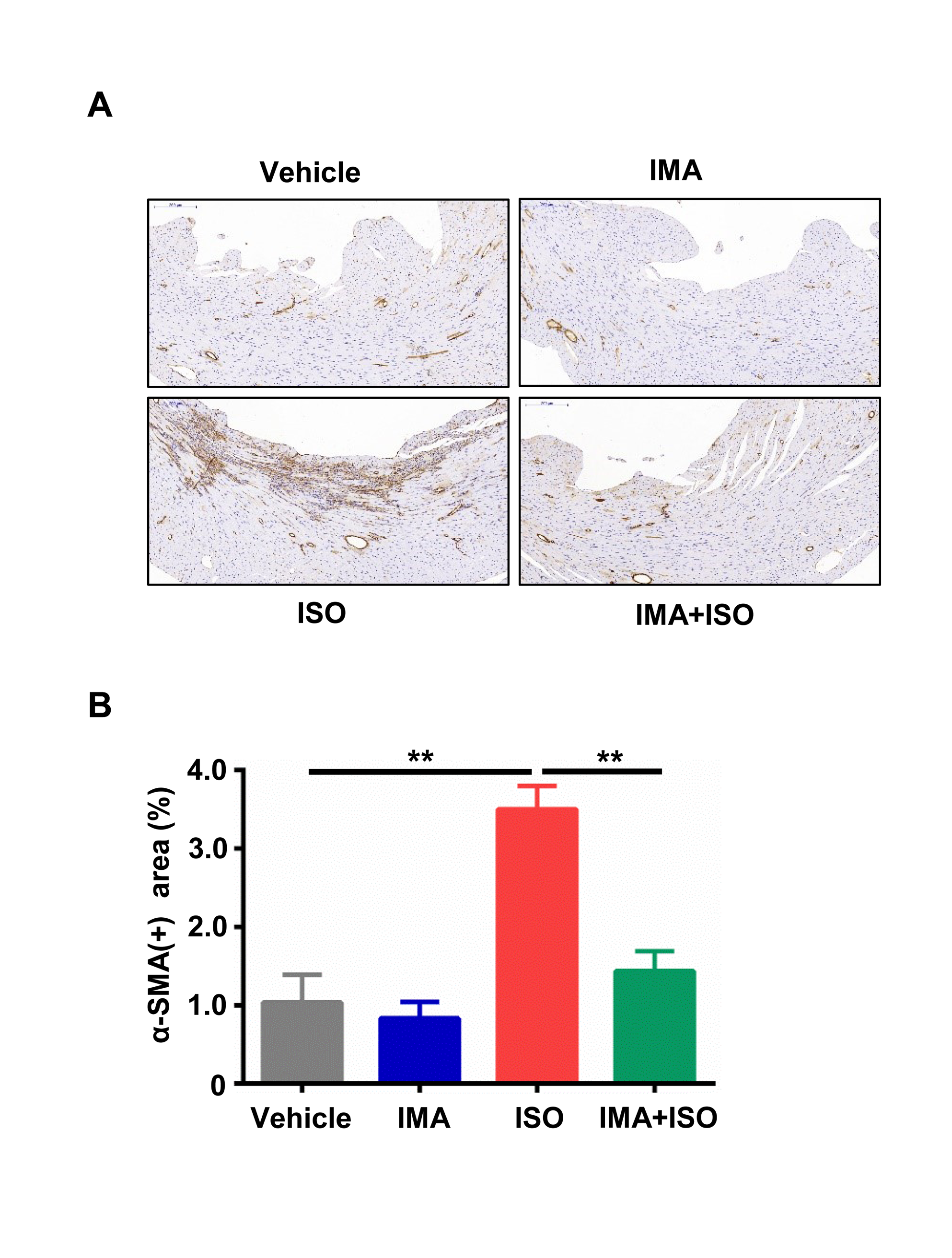

Supplement: S2 Fig — (A) (B) Histopathological feature of α-SMA in hearts was tested by immunohistologic analysis (100×) and quantitative analysis. (n = 8 per group, *: p<0.05, **: p<0.01). (TIF) [file pone.0178619.s002.tif]

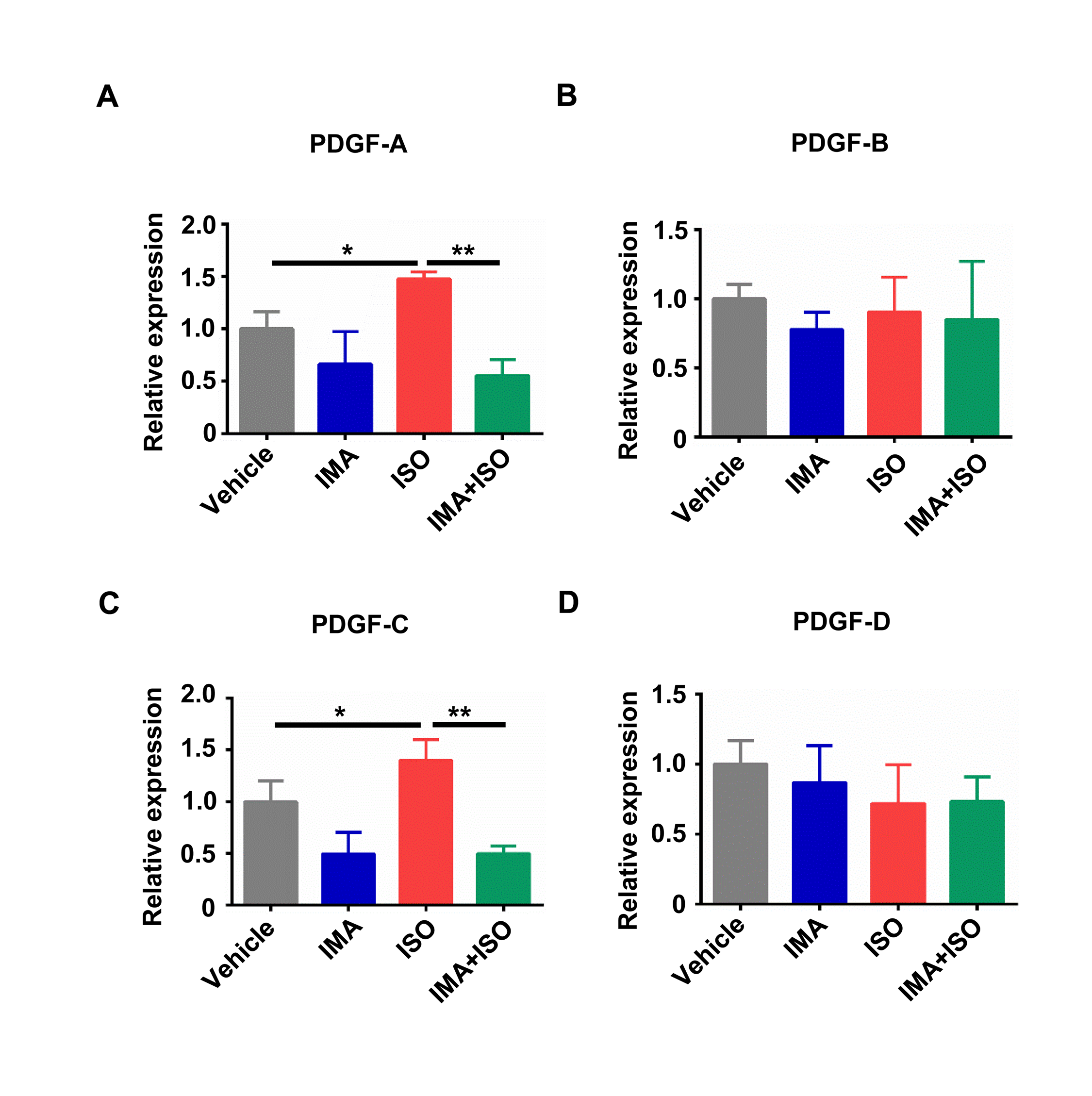

Supplement: S3 Fig — (A)-(D) The mRNA expression of PDGF-A, PDGF-B, PDGF-C, and PDGF-Din hearts from mice treated with vehicle, IMA, ISO, IMA + ISO for one week was tested by Western blot (n = 5–8 per group, *: p<0.05, **: p<0.01). (TIF) [file pone.0178619.s003.tif]
